# Protocol for a prospective, multicenter, randomized, controlled trial comparing pulsed field ablation vs. cryoballoon ablation in patients with persistent atrial fibrillation (PEACE trial)

**DOI:** 10.1016/j.ijcha.2025.101819

**Published:** 2025-10-07

**Authors:** Hidehira Fukaya, Jun Oikawa, Tomoharu Yoshizawa, Akira Satoh, Wataru Shinkai, Megumi Toraiwa, Sho Ogiso, Daiki Saito, Gen Matsuura, Shuhei Kobayashi, Yuki Arakawa, Hironori Nakamura, Naruya Ishizue, Jun Kishihara, Junya Ako

**Affiliations:** a Department of Cardiovascular Medicine, Kitasato University School of Medicine, Kanagawa, Japan; b Department of Kitasato Clinical Research Center, Kitasato University School of Medicine, Kanagawa, Japan; c Department of Cardiovascular Medicine, Sagamihara Kyodo Hospital, Kanagawa, Japan; d Department of Cardiovascular Medicine, Nerima Hikarigaoka Hospital, Tokyo, Japan

**Keywords:** Atrial fibrillation, Catheter ablation, Pulsed-field ablation, Cryoballoon ablation, Randomized controlled trial

## Abstract

**Background:**

Catheter ablation has become a standard treatment for atrial fibrillation (AF). However, evidence regarding the efficacy and safety of pulsed field ablation (PFA) in patients with persistent AF (PeAF) remains limited. The PEACE trial aims to evaluate the efficacy and safety of PFA compared to cryoballoon ablation (CBA) in PeAF.

**Methods:**

This prospective, multicenter, open-label, randomized controlled, non-inferiority trial (NCT07064616, UMIN000057896) will enroll 300 patients with PeAF, randomly assigned (1:1) to undergo either PFA using the PulseSelect™ or cryoballoon ablation (CBA) using the Arctic Front Advance™. The primary efficacy endpoint is atrial tachyarrhythmia recurrence within 12 months. The primary safety endpoint is procedure-related complications within 30 days. Secondary outcomes include early recurrence, changes in LA diameter, natriuretic peptide levels, and patient-reported symptoms.

**Expected results:**

We hypothesize that PFA will be non-inferior to CBA in terms of efficacy and safety.

**Conclusions:**

The PEACE trial will provide essential data regarding the efficacy and safety of PFA compared to CBA in patients with PeAF, potentially informing future clinical practice.

## 1 Introduction

Atrial fibrillation (AF) is the most common sustained tachy-arrhythmia worldwide and is associated with increased morbidity and mortality [1]. Catheter ablation has been established as an effective rhythm control strategy and is currently the first-line treatment for paroxysmal AF [[1], [2], [3]]. However, complications and recurrences remain concerns. Since the early 2000 s, radiofrequency (RF) ablation has been the dominant energy source. Cryoballoon ablation (CBA), introduced in 2014, enables standardized pulmonary vein isolation (PVI) with reduced operator-dependent variables [4,5]. The high durability of PVI using CBA was demonstrated [5,6], and CBA has been approved for use in patients with persistent AF (PeAF) [7].

More recently, pulsed field ablation (PFA) has emerged as a non-thermal, tissue-selective modality minimizing collateral damage [8]. However, data remain limited, particularly in PeAF. The PulseSelect™ PFA system (Medtronic, Minneapolis, MN, USA), an over-the-wire, circular PFA catheter, was introduced in 2024 [9] and is currently approved for PeAF. However, data on its efficacy and safety in PeAF remain scarce. Therefore, the PEACE (PulsEd field Ablation versus Cryoballoon ablation in patients with pErsistent atrial fibrillation) aims to compare the clinical efficacy and procedural safety of PFA (PulseSelect™) and CBA in patients with PeAF.

## 2 Methods

### 2.1 Study design

**Fig. 1:**
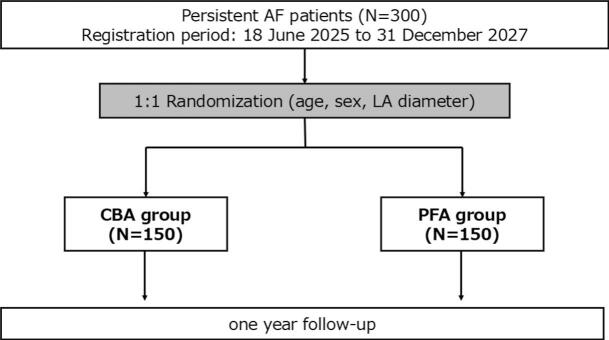
Study design and randomization process of the PEACE trial. All patients will be randomly assigned in a 1:1 ratio to either the PFA or the CBA group.

The PEACE trial is a prospective, multicenter, open-label, randomized controlled non-inferiority trial conducted at three hospitals in Japan. Patients will be randomly assigned in a 1:1 ratio to undergo either PFA or CBA (Fig. 1). The study aims to enroll 300 patients (150 in each group). Stratified randomization will be conducted using a random number table, based on age (<70 or ≥70 years), sex, and left atrial (LA) diameter (<40 mm or ≥40 mm), to ensure balanced allocation across these variables. Only one investigator (J.O.), who will not be involved in assigning the actual interventions, will have access to the randomization sequence. The planned enrolment period is from 18 June 2025 (the IRB-approved start date) to 31 December 2027.

### 2.2 Study population

#### 2.2.1 Inclusion criteria

* Age between 18 and 85 years.
* Diagnosed with PeAF (lasting less than one year or requiring cardioversion).
* Eligible for catheter ablation according to current Japanese guidelines [2,10].
* Able to provide written informed consent.

#### 2.2.2 Exclusion Criteria

* Diagnosis of Paroxysmal or long-standing persistent AF (defined as duration > 1 year).
* History of prior catheter ablation or surgical ablation for AF.
* Left atrial diameter > 55 mm.
* Known contraindications to contrast agents (e.g., bronchial asthma, renal impairment, or contrast allergy).
* Considered unsuitable participation by the investigators.

### 2.3 Ablation procedures

All patients will undergo a standard pre-procedural evaluation. Continuation or discontinuation of antiarrhythmic drugs prior to enrollment will be at the discretion of the treating physicians. Anti-arrhythmic drugs will be discontinued for at least five half-lives of each drug before the index CA. Ablation procedures will be performed using CE-marked PFA or CBA systems as part of routine clinical care. All ablation procedures will be performed under general anesthesia, using propofol, dexmedetomidine, fentanyl, and a neuromuscular blocker. Respiratory support will be provided via a laryngeal mask airway (igel®, Intersurgical Ltd., Wokingham, Berkshire, UK) with mechanical ventilation. Following a single *trans*-septal puncture using 8.5Fr SL-0 sheath (Abbott, St Paul, MN, USA), substrate mapping will be created using a high-density mapping catheter (HD-Grid®, Abbott, St. Paul, MN, or Octaray®, Biosense Webster, Diamond Bar, CA) with three-dimensional mapping system (EnSite X®, Abbott, St. Paul, MN, or CARTO 3®, Biosense Webster, Diamond Bar, CA) to enhance catheter localization and minimize fluoroscopic time. Before the Pulsed-field or cryo application, atropine is administered unless contraindicated.

The PulseSelect™ PFA system (Medtronic, Minneapolis, MN) will be used exclusively in the PFA group. Following pre-mapping, the SL-0 sheath will be exchanged for a 10Fr FlexCath Contour™ (Medtronic, Minneapolis, MN), after which the PulseSelect™ catheter will be introduced. Pulsed field energy will be delivered in four trains per application as the standard setting. The total number of PFA applications will be at the discretion of operators.

The Arctic Front Advance™ cryoballoon (Medtronic, Minneapolis, MN) will be used exclusively in the CBA group for this study. Similarly, after pre-mapping, SL-0 sheath will be exchanged for 12Fr FlexCath Contour™. Once the cryoballoon is inserted, cryo-application will be delivered to each PV. Application time will be set to 180–240 s per pulmonary vein, or 120 s after confirmation of PV isolation. An esophageal temperature monitoring catheter (Esophaster®, Japan Lifeline, Tokyo, Japan) will be inserted in all CBA group patients to minimize the risk of esophageal injury. If the temperature falls below 15 °C, the cryo-application will be immediately terminated.

After the treatment, post-mapping will be performed using a high-density mapping catheter to confirm the PVI and delineate the lesion area.

If atrial flutter, atrial tachycardia, or immediate recurrence of AF (IRAF) will occur or be induced during the procedure, additional ablation can be performed at the operator’s discretion. Any additional prophylactic ablation will not be allowed. All such events and treatments will be documented for subsequent analysis.

All procedures will be performed by the physicians who have at least 50 AF ablation cases annually and also have experience using PulseSelect™ and Arctic Front Advance™ Cryoballoon system in at least 10 cases before this study. All participating centers performed more than 150 AF ablations per year.

### 2.4 Follow-up data collection

Complications will be assessed as safety outcomes. Incidence of cardiac tamponade, transient/permanent phrenic nerve palsy, esophageal injury, and stroke events will be documented. Coronary spasm events will be documented during the procedure; if suspected, coronary angiography will be performed and vasodilator therapy administered. Groin hematoma or other bleeding complications after the procedure will also be assessed within 30 days after the index procedure. These events will be recorded as predefined safety endpoints to ensure consistent adjudication across participating centers.

Patient-reported symptom burden will be assessed using the Numeric Rating Scale (NRS: range 0–10) on the day following the index ablation. Since hemolysis and hemolysis-related renal insufficiency were reported in patients treated with PFA, laboratory parameters, including hemoglobin, creatinine kinase, serum creatinine, and urinary hemoglobin, will be measured at baseline, post-procedure day 1, at 6 and 12 months. Echocardiography and Holter monitoring will also be performed at 6 and 12 months. Seven-day Holter ECG monitoring is mandatory at each time point to evaluate the recurrence. Standard 12-lead ECGs, 24-hour or 7-day Holter ECG monitoring can also be performed at the physician’s discretion on other patient visits. Arrhythmia recurrence is defined as any atrial arrhythmia lasting ≥ 30 s, occurring after a 90-day blanking period, and detected by either 12-lead or Holter monitoring.

### 2.5 Study endpoints

✔ Primary efficacy endpoint.
  • Freedom from any atrial arrhythmia (AF, atrial flutter, or atrial tachycardia) recurrence lasting ≥ 30 s after a 90-day blanking period for 12 months post-ablation
✔ Primary safety endpoint.
  • Incidence of procedure-related complications within 30 days post-procedure
✔ Secondary endpoints:
  • Changes in LA diameter
  • Changes in BNP/NT-proBNP levels
  • Early arrhythmia recurrence (within the 90-day blanking period)
  • Patient-reported symptoms assessed using the NRS scale

### 2.6 Sample size calculation

The sample size of 300 patients (150 per group) was calculated based on an assumed 12-month success rate of 54.8 % for CBA vs. 55.1 % for PulseSelect™ for PeAF as reported in previous studies [7,9]. The calculation assumed a significance level (α) of 0.05, statistical power of 80 %, and a non-inferiority margin of 1.4. For safety endpoints, a complication rate of 4.9 % was estimated for both groups. To account for potential dropouts and missing data, approximately 10 % more patients were added, resulting in 150 patients per group.

### 2.7 Statistical analysis

Time-to-event analyses will be conducted using Kaplan-Meier survival curves and compared using the log-rank test. Categorical variables will be compared using the Chi-square test or Fisher’s exact test, while continuous variables will be analyzed using the Student’s *t*-test or the Wilcoxon rank-sum test, as appropriate. A two-sided p-value of <0.05 will be considered statistically significant.

Analyses will be conducted using all available data. Missing primary outcome data will be treated as censored observations in the time-to-event analysis rather than excluded. No imputation is planned. For secondary outcomes, available-case analysis will be performed, incorporating all observed data to minimize data loss. Sensitivity analyses may be considered if the extent of missing data is substantial.

### 2.8 Ethics

This study will be conducted in accordance with the principles of the Declaration of Helsinki. This protocol has been approved by the Ethics Committee of Kitasato University Hospital (Approval No. C24-157) and registered with both ClinicalTrials.gov (NCT07064616) and the University Hospital Medical Information Network (UMIN000057896). Written informed consent will be obtained from all participants prior to enrolment. The results will be submitted for publication in peer-reviewed journals and presented at relevant scientific conferences.

## 3 Discussion

The PEACE trial is the first randomized controlled trial to evaluate PFA using the PulseSelect™ in comparison with CBA using the Arctic Front Advance™ for patients with PeAF. The trial aims to provide critical evidence on whether PFA can achieve comparable efficacy to CBA while offering a reduced risk of complications.

Pulsed-field ablation was introduced into clinical practice in 2019. Its most distinctive feature is the tissue-selectivity of the pulsed-field energy [8]. PV stenosis is one of the serious complications associated with thermal energy-based catheter ablation procedures [11]. As of 2025, no cases of PV stenosis have been reported with PFA [12]. Furthermore, collateral damage such as esophageal ulcers, gastric hypomotility, and permanent phrenic nerve injury has not been observed. Although these complications have decreased even with the use of CBA, fatal events have still been reported. On the other hand, complications specific to PFA, such as hemolysis, hemolysis-related renal dysfunction [13], and coronary spasm [14], have been reported. This study will specifically assess these complications in comparison with those associated with thermal energy-based ablation systems. In this study, we will assess the incidence of hemolysis and related renal insufficiency via laboratory parameters (hemoglobin, creatine kinase, serum creatinine, and urinary hemoglobin) at baseline, day 1, 6, and 12 months. Moreover, the long-term efficacy of the PFA system remains insufficiently established, particularly in patients with PeAF. While thermal ablation typically produces an irreversible lesion, electroporation induced by pulsed-field energy has been reported to be partially reversible [15]. It remains challenging to determine whether the pulsed-field applications reliably create irreversible lesions, as local potential often disappears temporarily, regardless of the reversibility of the electroporation. Therefore, the durability of the created lesion following PFA will be a critical outcome of interest. A retrospective study comparing the recurrence of AF after three different energy sources for PeAF demonstrated [16] that penta-spline PFA and Arctic Front Advance™ cryoablation achieved compatible outcomes, outperforming radiofrequency catheter ablation.

This study has some limitations. Because it is an open-label design, blinding of patients and operators to the modality and outcomes is not feasible, which may introduce bias. Nonetheless, recurrence of atrial tachyarrhythmia will be adjudicated using objective evidence from ECGs or one-week Holter monitoring, which is expected to minimize its influence on the results.

The PEACE trial, as a prospective, randomized controlled trial, will evaluate the efficacy of PFA in comparison with CBA, which has demonstrated high lesion durability. This trial is expected to provide clinically meaningful evidence regarding the selection of energy sources for the ablation in patients with PeAF.

## 4 Conclusion

The PEACE trial is expected to provide pivotal data on the efficacy and safety of PFA using the PulseSelect™ compared with CBA in patients with PeAF. The findings will help inform future clinical practice and guide optimal energy source selection for catheter ablation in AF.

## Ethics approval statement

This study was approved by the ethics board at Kitasato University Hospital (No. C24-157).

**Clinical trial registration:** NCT07064616, UMIN000057896.

## CRediT authorship contribution statement

**Hidehira Fukaya:** Writing – original draft, Data curation, Conceptualization. **Jun Oikawa:** Writing – review & editing, Formal analysis, Conceptualization. **Tomoharu Yoshizawa:** Writing – review & editing, Data curation, Conceptualization. **Akira Satoh:** Writing – review & editing, Data curation, Conceptualization. **Wataru Shinkai:** Data curation. **Megumi Toraiwa:** Data curation. **Sho Ogiso:** Data curation. **Daiki Saito:** Data curation. **Gen Matsuura:** Data curation. **Shuhei Kobayashi:** Data curation. **Yuki Arakawa:** Data curation. **Hironori Nakamura:** Data curation. **Naruya Ishizue:** Data curation. **Jun Kishihara:** Data curation. **Junya Ako:** Writing – review & editing, Supervision.

## Funding

This research received no funding from any public, commercial, or not-for-profit agency.

## Declaration of competing interest

The authors declare the following financial interests/personal relationships which may be considered as potential competing interests: [Hidehira Fukaya reports a relationship with Medtronic Japan Co Ltd that includes: speaking and lecture fees. If there are other authors, they declare that they have no known competing financial interests or personal relationships that could have appeared to influence the work reported in this paper].

## Data Availability

Not applicable.
